# Metabolite identification through multiple kernel learning on fragmentation trees

**DOI:** 10.1093/bioinformatics/btu275

**Published:** 2014-06-11

**Authors:** Huibin Shen, Kai Dührkop, Sebastian Böcker, Juho Rousu

**Affiliations:** ^1^Department of Information and Computer Science, Aalto University, Espoo, Finland, ^2^Helsinki Institute for Information Technology, Espoo, Finland and ^3^Chair for Bioinformatics, Friedrich Schiller University Jena, Jena, Germany

## Abstract

**Motivation:** Metabolite identification from tandem mass spectrometric data is a key task in metabolomics. Various computational methods have been proposed for the identification of metabolites from tandem mass spectra. Fragmentation tree methods explore the space of possible ways in which the metabolite can fragment, and base the metabolite identification on scoring of these fragmentation trees. Machine learning methods have been used to map mass spectra to molecular fingerprints; predicted fingerprints, in turn, can be used to score candidate molecular structures.

**Results:** Here, we combine fragmentation tree computations with kernel-based machine learning to predict molecular fingerprints and identify molecular structures. We introduce a family of kernels capturing the similarity of fragmentation trees, and combine these kernels using recently proposed multiple kernel learning approaches. Experiments on two large reference datasets show that the new methods significantly improve molecular fingerprint prediction accuracy. These improvements result in better metabolite identification, doubling the number of metabolites ranked at the top position of the candidates list.

**Contact:** huibin.shen@aalto.fi

**Supplementary information:** Supplementary data are available at *Bioinformatics* online.

## 1 INTRODUCTION

Metabolomics deals with the analysis of small molecules and their interactions in living cells. A central task in metabolomics experiments is the identification and quantification of the metabolites present in a sample. This is mandatory for subsequent analysis steps such as metabolic pathway analysis and flux analysis (Pitkänen *et al.*, 2010). Mass spectrometry (MS) is one of the two predominant analytical technologies for metabolite identification. Identification is done by fragmenting the metabolite, for example, by tandem MS (MS/MS), and measuring the mass-to-charge ratios of the resulting fragment ions. The measured mass spectra contain information about the metabolite, but extracting the relevant information is a highly non-trivial task.

Several computational methods have been suggested to identify the metabolites from MS/MS spectra. Mass spectral databases (spectral libraries) have been created (e.g. Hisayuki *et al.*, 2010; Oberacher *et al.*, 2009; Smith *et al.*, 2005; Tautenhahn *et al.*, 2012), which allow us to search measured mass spectra. Unfortunately, this approach can only identify ‘known unknowns’ where a reference measurement is available.

Fragmentation trees are combinatorial models of the MS/MS fragmentation process. Böcker and Rasche (2008) suggested fragmentation trees for identifying the molecular formula of an unknown compound. Later, fragmentation trees were shown to contain valuable structural information about the compound (Rasche *et al.*, 2011, 2012).

The relation between spectral and structural similarities has been studied by Demuth *et al.* (2004). A kernel-based machine learning approach for metabolite identification was recently introduced by Heinonen *et al.* (2012), relying on predicting the molecular fingerprints as an intermediate step. Molecular fingerprints are given as bit vectors with each bit describing the existence of certain molecular property such as substructures in the molecule. After the prediction, imposing some scoring strategy, the predicted molecular fingerprints are used for searching some chemical database and finally the ranked list of candidates are generated (Heinonen *et al.*, 2012; Shen *et al.*, 2013).

Besides these two approaches, methods have been suggested for predicting MS/MS spectra from molecular structures (Allen *et al.*, 2013; Kangas *et al.*, 2012); commercial software packages also exist for this task. Such simulated spectra can be used to replace the notoriously incomplete spectral libraries by molecular structure databases (Hill *et al.*, 2008). Combinatorial fragmentation of molecular structure serves the same purpose (Gerlich and Neumann, 2013; Wolf *et al.*, 2010). Finally, we can search spectral libraries for similar compounds, by comparing either MS/MS spectra (Demuth *et al.*, 2004; Gerlich and Neumann, 2013) or fragmentation trees (Rasche *et al.*, 2012). See Scheubert *et al.* (2013) and Hufsky *et al.* (2014) for recent reviews.

We propose a joint strategy that combines fragmentation trees and multiple kernel learning (MKL) to improve molecular fingerprint prediction and, subsequently, the metabolite identification. We first outline the metabolite identification framework and introduce fragmentation trees and their computation. Next, we introduce a family of kernels for fragmentation trees, consisting of simple node and edge statistics kernels as well as path and subtree kernels that use dynamic programming (DP) for efficient computation. We then describe state-of-the-art methods for MKL. In these experiments, we evaluate different MKL algorithms with regards to the fingerprint prediction and the metabolite identification.

## 2 METHODS

**Fig. 1.**
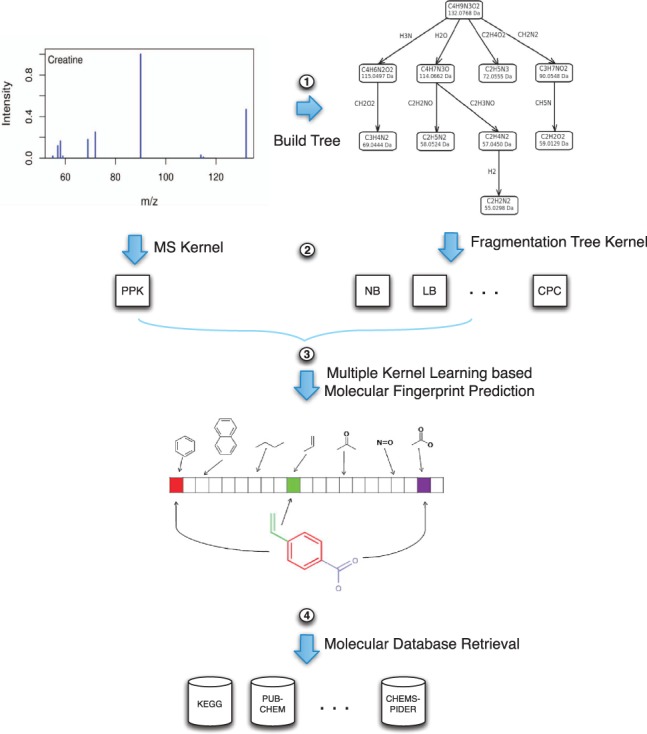
The metabolite identification framework through MKL. First, we construct the fragmentation tree from the MS/MS spectrum. Second, we compute kernels for both MS/MS data and fragmentation trees. Third, MKL is used to combine kernels and predict molecular fingerprints. Finally, fingerprints are used for molecular structure database retrieval

Figure 1 gives an overview for our metabolite identification framework through MKL. Fragmentation trees are computed first, followed by the computation of kernels. MKL approaches are used to integrate different kernels for molecular fingerprint prediction. The final step of the framework is to query molecular structure databases with the predicted molecular fingerprint using a probabilistic scoring function.

The advantages of the kernel-based machine learning framework are: that it easily allows incorporating the combinatorial fragmentation trees by kernelizing the model; that it can query molecular structure databases which are much larger than MS/MS spectral libraries; and that molecular fingerprints can help to characterize the unknown metabolite and may shed light for *de novo* identification.

### 2.1 Fragmentation trees

Böcker and Rasche (2008) introduced fragmentation trees to predict the molecular formula of an unknown compound using its MS/MS spectra. A fragmentation tree annotates the MS/MS spectra of a compound via assumed fragmentation processes. Nodes are molecular formulas, representing the unfragmented molecule and its fragments. Edges represent fragmentation reactions between fragments, or the unfragmented molecule and a fragment. Details on the computation can be found in Böcker and Rasche (2008) and Rasche *et al.* (2011); here, we quickly recapitulate the method. We assume that MS/MS spectra recorded at different collision energies have been amalgamated into a single spectrum, as described in Section 3. We decompose all peaks in the amalgamated spectrum, finding all molecular formulas that are within the mass accuracy of the measurement. For each decomposition of the parent peak, we build a fragmentation graph which contains all possible explanations for each peak, where nodes are colored by the peaks they originate from. We insert all edges between nodes that are not ruled out by the molecular formulas: that is, a product fragment can never gain atoms of any element through the fragmentation. Edges of this graph are then weighted, taking into account the intensity and mass accuracy of the product fragment, the mass of the loss and prior knowledge about the occurrence of certain losses.

Under the parsimony assumption, we then compute a colorful subtree of this graph with maximum weight. Unfortunately, finding this tree is an NP-hard problem (Rauf *et al.*, 2012). Nevertheless, we can compute optimal trees in a matter of seconds using Integer Linear Programming (Rauf *et al.*, 2012). For each peak, this tree implicitly decides whether it is noise or signal and, in the later case, assigns the molecular formula of the corresponding fragments and the fragmentation reaction it resulted from. The score of the tree is the sum of its edge weights. Candidate molecular formulas of the parent peak are ranked by this score, which is the maximum score of any tree that has this molecular formula as its root.

Different from Böcker and Rasche (2008) and Rasche *et al.* (2011), we used a modified weighting function for the edges of the fragmentation graph. With these new weights, the above optimization can be interpreted as a maximum a posteriori estimator of the observed data. We weight edges by the logarithmic likelihood that a certain fragmentation reaction occurs: for this, we consider the intensity and mass deviation of the product fragment peak, the loss mass and chemical properties of the molecular formula as proposed in Kind and Fiehn (2007): namely, the ring double bond equivalent and the hetero atoms and carbon atoms ratio. Furthermore, we favor a few common losses that were learned from the data, and penalize implausible losses and radicals. Such weights have already been used in Böcker and Rasche (2008) and Rasche *et al.* (2011); different from there, we did not choose parameters *ad hoc* but rather learned them from the data. Details about these new weights will be published elsewhere.

### 2.2 Kernels for fragmentation trees and MS/MS spectra

#### 2.2.1 Probability product kernel

Heinonen *et al.* (2012) compared several kernels that can be computed directly from the MS/MS spectra without the knowledge of the fragmentation trees. In their studies, simple peak and loss matching kernels were found inferior to the probability product kernel (PPK). Thus, we use the PPK as the baseline comparison with the fragmentation tree kernels. The idea of the PPK is the following: each peak in a spectrum is modeled by a 2D Gaussian distribution with the mass-to-charge ratio as one dimension, and the intensity as the other. All-against-all matching between the Gaussians is performed to avoid problems arising from alignment errors.

Formally, a spectrum is defined as $\chi =\{\chi (1),\ldots ,\chi (\ell_{\chi })\}$, a set of *ℓ*_χ_ peaks $\chi (k)=\left(\mu (k),\iota (k)\right)\in {\mathbb{R}}^{2},(k=1,\ldots ,\ell_{\chi })$ consisting of the peak mass μ(*k*) and the normalized peak intensity ι(*k*). The *k*-th peak of the mass spectrum χ is represented by $p_{\chi (k)}=N(\chi (k),\Sigma )$ centered around the peak measurement and with covariance shared with all peaks

$$\Sigma =\left[\begin{array}{cc} \sigma_{\mu }^{2} & 0\\ 0 & \sigma_{\iota }^{2} \end{array}\right]$$

where the variances $\sigma_{\mu }^{2}$ for the mass is estimated from data and $\sigma_{\iota }^{2}$ is tuned by cross-validation. No covariance is assumed between peak distributions. The spectrum χ is finally represented as a mixture of its peak distributions $p_{\chi }=\frac{1}{\ell_{\chi }}\sum_{k=1}^{\ell_{\chi }}p_{\chi (k)}$.

The PPK *K*_peaks_ (Jebara *et al.*, 2004) between the peaks of two spectra χ, χ′ is given by:

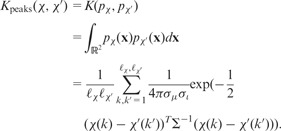



The precursor ion is the compound selected in the first round of MS/MS and further fragmented in the second round. As a result, the difference (loss) between the peak χ(*k*) and the precursor ion prec(χ) = (μ(*p*),0) is also important, where *μ*(*p*) is the mass of the precursor ion. We can model the difference with distribution $p_{\hat{\chi }(k)}=N(\hat{\chi }(k),\Sigma )$, where $\hat{\chi }(k)=|\text{prec}(\chi )-\chi (k)|$. This feature is denoted as loss and corresponding kernel matrix as *K*_loss_. Experiments in Heinonen *et al.* (2012) and Shen *et al.* (2013) showed that the combined kernel *K*_peaks_ + *K*_loss_ achieved best accuracy and computational efficiency among the spectral kernels.

#### 2.2.2 Fragmentation tree kernels

Fragmentation trees can be considered as an annotated representation of the original MS/MS spectra. Recent advancement (Rasche *et al.*, 2012; Rojas-Chertó *et al.*, 2012) in comparing and aligning the fragmentation trees enables similarity metrics to be defined between fragmentation patterns for small molecules. Rasche *et al.* (2012) introduced fragmentation tree alignments, and showed alignment scores to be correlated with chemical similarity. However, alignment scores of this type do not, in general, yield positive semidefinite kernels. In the following, we define a set of kernels for fragmentation trees that will allow us to transfer the power of the fragmentation tree approach to the kernel-based learning algorithms for molecular fingerprint prediction and metabolite identification.

A fragmentation tree *T* = (*V*, *E*) consists of a nodes set *V* of molecular formulas (corresponding to the fragments) and an edges set *E* ⊆ *V* × *V* (corresponding to the losses). Let *r* denote the root of *T*. For an edge e=(u,v)∈E let λ(e)=λ(u,v):=u−v be the molecular formula of the corresponding loss. Clearly, different edges may have identical losses; let λ(*E*) be the multiset of all losses. For some loss molecular formula *l*, let *N*(*l*) be the number of edges e∈E with λ(*e*) = *l*. Each path from the root *r* to a node *v* implies a root loss *r* − *v*; let E:={r−v:v∈V} be the set of all root losses. For a MS/MS spectrum *x*, let *T_x_* = (*V_x_*,*E_x_*) be the corresponding fragmentation tree, with root losses $E_{x}$ and loss multiplicities *N_x_*(·). For any node $v\in V_{x}$ let ι*_x_*(*v*) be the corresponding peak intensity; for an edge $e=(u,v)\in E_{x}$ let ι*_x_*(*e*) be the intensity of the terminal node *v*.

For the loss- and node-based kernels, feature vectors ϕ are constructed and the kernel function is just a simple dot product between two feature vectors. Path-based kernels are more complicated, and details on their computation will be given below.

*Loss-based kernels*: edges in the fragmentation trees represent the losses from the parent node to the child node. The following feature vectors are devised based on the losses in a fragmentation tree *T_x_*:

- LB: Loss binary, indicates the presence of a loss *l* in a fragmentation tree *T_x_*, that is, 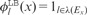.
- LC: Loss count, counts the number of occurrences of a loss *l* in a fragmentation tree *T_x_*, that is, $\phi_{l}^{\mathrm{LC}}(x)=N_{x}(l)$.
- LI: Loss intensity, uses the average intensity of the terminal nodes with loss *l* in a fragmentation tree *T_x_*, that is, $\phi_{l}^{\mathrm{LI}}(x)=\frac{1}{N_{x}(l)}\sum_{e\in E_{x}\lambda (e)=l}\iota_{x}(e)$.
- RLB: Root loss binary, indicates the presence of a root loss *l* in a fragmentation tree *T_x_*, that is, $\phi_{l}^{\mathrm{RLB}}(x)=1_{l\in E_{x}}$.
- RLI: Root loss intensity uses the intensity of the terminal node of a root loss if it is present in a fragmentation tree *T_x_*. For root *r* we set $\phi_{l}^{\mathrm{RLI}}(x)=\iota_{x}(r-l)$ if $r-l\in V_{x}$, and $\phi_{l}^{\mathrm{RLI}}(x)=0$ otherwise.

*Node-based kernels*: the nodes in the fragmentation tree explain peaks in the MS/MS by some chemical formula of the hypothetical fragment. The nodes are unique in a fragmentation tree *T*, and so are the root losses. To this end, we can omit root losses from the feature vectors.

- NB: Nodes binary, indicates the presence of a node *v* in a fragmentation tree *T_x_*, that is, $\phi_{v}^{\mathrm{NB}}(x)=1_{v\in V_{x}}$.
- NI: Nodes intensity, uses the intensity of the node if it is presented in a fragmentation tree *T_x_*; that is, $\phi_{v}^{\mathrm{NI}}(x)=\iota_{x}(v)$ for $v\in V_{x}$, and $\phi_{v}^{\mathrm{NI}}(x)=0$ otherwise.

*Path-based kernels*: these kernels are count common path between two fragmentation trees—here, ‘common path’ refers to an identical sequence of losses in the two trees. We use DP to efficiently count the number of common paths, that is, the dot product of two feature vectors which are not explicitly constructed. For two fragmentation trees *T*_1_ = (*V*_1_,*E*_1_) and *T*_2_ = (*V*_2_,*E*_2_) we compute a DP table *D*[*u*,*v*] for all $u\in V_{1}$ and $v\in V_{2}$. In all cases, the number of common paths is *D*[*r*_1_,*r*_2_] where *r_i_* is the root of *T_i_*. We initialize

$$\begin{array}{l} D[u,v]=0,\forall u\in L(T_{1}),v\in T_{2}\\ D[u,v]=0,\forall u\in T_{1},v\in L(T_{2}) \end{array}$$

where L(T) denotes the leaves of a tree *T*. Let *C*(*v*) be the children of a node *v*.

- Common path counting (CPC). The DP table entry *D*[*u*,*v*] records the count of common path for the subtrees rooted in *u* and *v*, respectively. This leads to the following recurrence:

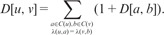

- Common paths of length 2 (CP2). In this case, only common losses for paths of length two are considered:

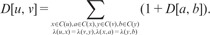

- Common path with *K*_peaks_ (CPK). Instead of simply counting the common paths, we use the PPK *K*_peaks_ to score the terminal peaks. We omit the straightforward but somewhat tedious details.
- Common subtree counting (CSC). In this case, we count the number of ‘common subtrees’ between *T*_1_ and *T*_2_, which can be defined analogously to the common paths above. Entry *D*[*u*,*v*] now counts the number of common subtrees for the two subtrees rooted in *u* of *T*_1_, and *v* of *T*_2_. We have to consider three cases: for each pair of children a∈C(u) and b∈C(v) with λ(*u*,*a*) = λ (*v*,*b*) we can either attach the subtrees rooted in *a* and *b*; we can use solely the edges (*u*, *a*) and (*v*, *b*) as a common subtree; or, we can attach no common subtree for this pair of children. But if we choose no subtree for all matching pairs of children, the result would be a tree without edges and, hence, not a valid common subtree. Thus, we have to correct for this case by subtracting one. Hence, the recurrence is:

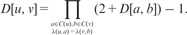

### 2.3 MKL

In many applications, multiple kernels from different kernel functions or multiple sources of information are available. MKL becomes a natural way to combine information contained in the kernels. Instead of choosing the best kernel via cross-validation as in Heinonen *et al.* (2012) and Shen *et al.* (2013), MKL seeks a linear, convex or even non-linear combination of the kernels. An overview of MKL algorithms can be found in a survey by Gönen and Alpaydin (2011).

In practice, it is often difficult for MKL algorithms to outperform the uniform combination of the kernels (UNIMKL) where the weights for kernels are equal. However, in some cases, some methods have seen improvements over the uniform combinations. Three algorithms coupled with SVM are considered in the following: centered alignment-based algorithms (Cortes *et al.*, 2012), quadratic combination of the kernels (Li and Sun, 2010) and *ℓ_p_*-norm *P* > 1 for the kernel weights (Kloft *et al.*, 2011).

For all the three algorithms, the input will be a set of kernels $K=\{K_{k}|K_{k}\in {\mathbb{R}}^{n\times n},k=1,\ldots ,q\}$ computed from *n* data points. The output is a set of *m* fingerprint properties $Y\in {\left\{-1,+1\right\}}^{n\times m}$ which is a multi-label prediction task and each label is trained independently in the experiments.

#### 2.3.1 Centered alignment-based MKL

The centered alignment-based MKL algorithms are based on the observation that the centered alignment score with the target kernel $K_{Y}=yy^{T}$ correlates very well with the performance of the kernel, where **y** is a single label. Experiments by Cortes *et al.* (2012) show consistent improvements over the uniform combination. In the molecular fingerprint prediction setting, the target kernel is defined as $K_{Y}=YY^{T}$.

Two-stage model are considered in which the kernel weights are learned first and then can be applied to all kernel-based learning algorithms (SVM in this work). The centered kernel matrices are defined by Equation (1):

(1) $$K_{c}=[I-\frac{ee^{T}}{n}]K[I-\frac{ee^{T}}{n}]$$

where **I** is the identity matrix and **e** is the vector with all ones. $\forall A,B\in {\mathbb{R}}^{n\times n}$, let ${\langle \cdot ,\cdot \rangle }_{F}$ denotes the Frobenius product and $||\cdot |{|}_{F}$ denotes the Frobenius norm which are defined by

$${\langle A,B\rangle }_{F}=Tr[A^{T}b]\;\;\text{and}\;\;||A|{|}_{F}=\sqrt[{}]{{\langle A,A\rangle }_{F}}.$$

Let now $K\in {\mathbb{R}}^{n\times n}$ and $K\prime \in {\mathbb{R}}^{n\times n}$ be two kernel matrices such that $||K_{c}|{|}_{F}\neq 0$ and $||K\prime_{c}|{|}_{F}\neq 0$. Then the centered alignment between **K** and **K***′* is defined by

(2) $$\hat{\rho }(K,K\prime )=\frac{{\langle K_{c},K_{c}\;\prime \rangle }_{F}}{||K_{c}|{|}_{F}||K\prime_{c}|{|}_{F}}.$$

The simple independent centered alignment-based algorithm (ALIGN) (Cortes *et al.*, 2012) computes the alignment score between each kernel matrix **K***_i_* and the target kernel matrix **K***_Y_* and combine the kernels as

$$\begin{array}{l} K_{\mu }\propto \sum_{k=1}^{q}\;\hat{\rho }(K_{k},K_{Y})K_{k}\\ \;=\frac{1}{||K_{Y}|{|}_{F}}\sum_{k=1}^{q}\;\frac{{\langle K_{k},K_{Y}\rangle }_{F}}{||K_{k}|{|}_{F}}K_{k}. \end{array}$$

The alignment maximization algorithm (ALIGNF) (Cortes *et al.*, 2012) jointly seeks the weight μ*_i_* to maximize the alignment score defined by Equation (2) between the convex combination of the kernel in **K** and the target kernel $K_{Y}=yy^{T}$, that is, the following optimization problem:

$$\max_{\mu \in M}\frac{{\langle K_{\mu },K_{Y}\rangle }_{F}}{||K_{\mu }|{|}_{F}}$$

where $M=\mu :||\mu |{|}_{2}=1,\mu \geq 0$.

#### 2.3.2 Quadratic combination MKL

In this setting, the quadratic combination of kernels (QCMKL) is included in the formulation and the MKL problem is solved by semidefinite programming (Lanckriet *et al.*, 2002; Li and Sun, 2010). The kernels in **K** are enriched to a new set $\tilde{K}=\{{\tilde{K}}_{t}|t=1,\ldots ,q(q+1)/2\}$ by the following transformation:

$${\tilde{K}}_{t(i,j)}=\{\begin{array}{cc} K_{i}\circ K_{j} & i\neq j\\ K_{i} & i=j \end{array}$$

where *i*,*j* = 1, … ,*q* and ∘ denotes the Hadamard product.

The convex combinations of the kernels is given by ${\tilde{K}}_{\mu }=\sum_{t=1}^{q(q+1)/2}\mu_{t}\tilde{K_{t}}$ with μ≥0 and $e^{T}\mu =1$. Adapting the soft margin SVM formulation reveals the following dual problem (in epigraph form) (Li and Sun, 2010):

$$\begin{array}{l} \max_{\alpha ,u}\;u\\ \text{s}.\text{t}.\;u\geq \alpha^{T}e\text{-}\frac{1}{\text{2}}\alpha^{T}G({\tilde{K}}_{\mu })\alpha ,\\ \;0\leq \alpha \leq Ce,\alpha^{T}y=0,\\ \;\mu \geq 0,e^{T}\mu =1. \end{array}$$

The derived Lagrangian for the problem is (Li and Sun, 2010):

$$\begin{array}{l} L(\alpha ,\beta ,\delta ,\gamma )=\alpha^{T}e-\frac{1}{2}\alpha^{T}G({\tilde{K}}_{\mu })\alpha +\beta^{T}\alpha \\ \;+\;\gamma \alpha^{T}y+\delta (Ce-\alpha ) \end{array}$$

with α,β≥0,δ≥0,γ as dual variables, and G(K)=diag(y)Kdiag(y). Applying Schur’s lemma to convert the first inequality constraint to Linear Matrix Inequality (LMI) unveils the following semidefinite program (SDP) (Li and Sun, 2010):

$$\begin{array}{l} \min_{\alpha ,u}\;u\\ \text{ s}.\text{t}.\;\left(\begin{array}{cc} G({\tilde{K}}_{\mu }) & e+\beta +\gamma y-\delta \\ {\left(e+\beta +\gamma y-\delta \right)}^{T} & u-2C\delta^{T}e \end{array}\right)≽0\\ \;\mu \geq 0,e^{T}\mu =1,\beta \geq 0,\delta \geq 0. \end{array}$$

Many standard SDP solvers can be used to find the optimal solutions such as cvx (http://cvxr.com/).

#### 2.3.3 ℓ_p_-norm MKL

While *ℓ*_1_ norm on the kernel weights *μ* produces sparse solutions, higher norms *p* > 1 produces non-sparse solutions which may be beneficial. A general framework for *ℓ_p_*-norm MKL (*ℓ_p_*-MKL) was proposed by Kloft *et al.* (2011). The *q* kernels correspond to *q* feature mappings $\Psi_{k}:\chi \to H_{k},k=1,\ldots ,q$ and *l* is some convex loss function and the primal problem is then:

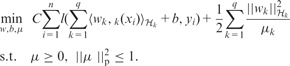

when the optimization is coupled with hinge loss, the problem has a simple dual form (Kloft *et al.*, 2011):

$$\max_{\alpha }\;\;\alpha^{T}e-\frac{1}{2}||{\left(\alpha^{T}G(\tilde{K_{i}})\alpha \right)}_{k=1}^{q}|{|}_{p^{*}},$$

where all the variables are all as defined before but $p^{*}=\frac{p}{p-1}$.

The optimization problem can be solved by alternating the dual variables *α* and the kernel weights *μ* via the squared norm on *w* by the following equations:

(3) $$||w_{k}|{|}^{2}=\mu_{k}^{2}\alpha^{T}K_{k}\alpha ,\forall k=1,\ldots ,q.$$

(4) $$\mu_{k}=\frac{||w_{k}|{|}^{2}}{{\left(\sum_{k\prime =1}^{q}||w_{k\prime }|{|}^{\frac{2p}{p+1}}\right)}^{\;\frac{1}{p}}},\forall k=1,\ldots ,q.$$

Based on the above equations, a simple alternating algorithm has been proposed by Kloft *et al.* (2011) as Algorithm 1.

**Algorithm 1 Wrapper algorithm for *ℓ_p_*-norm MKL**

Input feasible *α* and *μ*

**while** optimization conditions are not satisfied **do**

 Solve α with current μ using standard SVM.

 Compute $||w_{k}|{|}^{2}$ with equation (3).

 Update μ by equation (4).

**end while**

The optimization conditions can be the difference of objective function or the duality gap between two subsequent iterations. More detailed, theoretical results and a faster chunking-based algorithm are also presented in Kloft *et al.* (2011).

### 2.4 Probabilistic scoring of candidate metabolites

Given a predicted fingerprint associated with a mass spectrum, for metabolite identification, we need to retrieve metabolites with similar fingerprints from a molecular database. Assume $\hat{y}\in {\left\{-1,+1\right\}}^{m}$ is a predicted fingerprint and an arbitrary fingerprint $y\in {\left\{-1,+1\right\}}^{m}$ for some molecule in some molecular database, one can score the **y** by the following equation as used in *FingerID* (Heinonen *et al.*, 2012; Shen *et al.*, 2013):

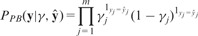

that is, the Poisson binomial probability for the fingerprint vector **y** where the cross-validation accuracies 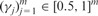 of the fingerprints prediction are taken as the reliability scores.

## 3 RESULTS

Two MS/MS datasets, 978 compounds downloaded from METLIN (Tautenhahn *et al.*, 2012) and 402 compounds from MassBank (Hisayuki *et al.*, 2010), both measured by QTOF MS/MS instruments are tested. For each compound, mass spectra recorded at different collision energies were amalgamated before further processing: we normalize MS/MS spectra such that intensities sum up to 100%. We merge peaks from different collision energies with *m*/*z* difference at most 0.1, using the *m*/*z* of the highest peak and summing up intensities. We discard all but the 30 highest peaks, as well as peaks with relative intensity <0.5%.

Next, we compute the fragmentation tree. We assume that we can identify the correct molecular formula from the data: limiting candidate molecular formulas to those present in KEGG (Kanehisa and Goto, 2000), which is used for searching molecular structures below, the best scoring fragmentation tree identified the correct molecular formula of the compound in 97.1% (96.0%) of the cases for the METLIN (MassBank) dataset. Integrating other sources of information such as MS1 isotope patterns (Böcker *et al.*, 2009) or retention times would reach even better identification rates. To allow for a meaningful comparison of the power of the different kernels, we therefore use the best scoring fragmentation tree of the correct compound molecular formula.

All 11 fragmentation tree kernels proposed in the previous section were computed, along with PPK used in Heinonen *et al.* (2012) and Shen *et al.* (2013) computed directly from MS/MS, resulting in 12 kernels to be evaluated.

Molecular fingerprints were generated using OpenBabel (O’Boyle *et al.*, 2011) which contains four types of fingerprints (http://openbabel.org/wiki/Tutorial:Fingerprints). FP3, FP4 and MACCS fingerprints (528 bits in total) were generated based on the software predefined SMARTS patterns. In our dataset, more than half of the fingerprint properties have high-class bias rate, with a large majority of the dataset belonging to the positive class (most compounds match the property) or respectively the negative class (most compounds do not match the property). For such fingerprints, the default classifier, one that always predicts the majority class, has high accuracy, although the model is not meaningful. For our performance comparisons, we opted to only include fingerprints with class bias rate <0.9.

For each fingerprint property, we separately trained a SVM; for all properties, we used identical training and testing compounds. Five-fold cross-validation was performed and the SVM margin softness parameter ($C\in \{2^{-3},2^{-2},\ldots ,2^{6},2^{7}\}$) was tuned based on the training accuracy.

### 3.1 Fingerprint prediction performance

The micro-average (simultaneous average over fingerprint properties and compounds) accuracy and F1 of the individual kernels on the predictions of fingerprint properties with bias rate <0.9 are shown in Table 1 with the SDs computed from different cross-validation folds. The kernel NB achieves the best accuracy and F1 on both METLIN and MassBank. Compared with the PPK, the fragmentation tree kernels are markedly more accurate on average.

**Table 1.** Micro-average performance of individual kernels

	METLIN		MassBank	
	Acc (%)	F1 (%)	Acc (%)	F1 (%)
LB	79.5 ± 0.5	69.9 ± 0.9	78.9 ± 1.0	69.0 ± 2.2
LC	79.4 ± 0.3	69.6 ± 0.4	78.5 ± 1.2	68.4 ± 2.7
LI	77.8 ± 0.5	66.8 ± 0.7	77.4 ± 1.0	66.7 ± 2.0
RLB	81.6 ± 0.8	73.2 ± 1.1	78.6 ± 1.0	68.4 ± 1.2
RLI	78.4 ± 0.6	68.5 ± 0.8	76.7 ± 0.9	65.4 ± 1.6
NB	**81.9 ± 0.4**	**73.9 ± 0.3**	**81.4 ± 0.7**	**73.2 ± 1.2**
NI	80.3 ± 0.7	71.1 ± 0.8	79.8 ± 1.0	70.5 ± 0.9
CPC	80.6 ± 0.5	71.6 ± 0.7	78.7 ± 1.4	68.9 ± 2.4
CP2	78.7 ± 0.7	68.4 ± 1.2	76.4 ± 1.0	65.5 ± 1.1
CPK	72.9 ± 0.3	58.8 ± 0.5	72.2 ± 0.6	57.9 ± 0.5
CSC	74.9 ± 0.4	61.9 ± 0.8	77.8 ± 0.8	67.2 ± 2.0
PPK	76.7 ± 0.6	64.0 ± 0.7	72.9 ± 1.1	58.6 ± 1.2

PPK is the method from Heinonen *et al.* (2012), which we compare against.

The improvement of MKL approaches over single kernel SVMs are clear. The *t*-test between NB and ALIGNF shows the differences of mean accuracy and F1 are indeed very significant with *P*-values of 4 × 10^−^^6^ and 1.7 × 10^−^^3^, respectively. The kernel weights learned by different MKL algorithms are shown in the supplementary file.

The micro-average accuracy and F1 of the MKL algorithms on the fingerprint properties predictions are shown in Table 2, where it can be concluded that averaged overall fingerprints of the MKL methods are quite close. We conducted further pairwise difference testing, where the performance difference of each method on each individual fingerprint property is evaluated. Table 3 shows the significance level of the sign test on the accuracy and F1 on the METLIN and MASSBANK datasets using the different MKL methods. The sign test describes whether one of the methods has higher probability of success (better than the other on a fingerprint) than the other (alternative hypothesis) or not (null hypothesis). From the table, we can deduce that ALIGN and ALIGNF rise slightly above the competition whereas *ℓ*_2_-MKL and QCMKL are slightly inferior to the rest. The performance of UNIMKL is also respectable. The scatter plots of accuracy and F1 between every pair of the MKL algorithms are shown in the supplementary file.

**Table 2.** Micro-average performance of MKL algorithms

	METLIN		MassBank	
	Acc (%)	F1 (%)	Acc (%)	F1 (%)
UNIMKL	85.0 ± 0.6	78.3 ± 0.7	82.2 ± 0.6	73.9 ± 1.5
ALIGN	**85.2 ± 0.6**	78.6 ± 0.7	82.4 ± 0.7	74.4 ± 1.4
ALIGNF	85.0 ± 0.5	**78.6 ± 0.4**	**82.8 ± 0.4**	**75.2 ± 1.2**
QCMKL	84.9 ± 0.5	77.8 ± 0.5	82.1 ± 0.6	74.0 ± 0.7
*ℓ*_2_-MKL	84.7 ± 0.5	77.5 ± 0.5	82.2 ± 0.5	74.0 ± 0.9
*ℓ*_3_-MKL	**85.2 ± 0.6**	78.5 ± 0.7	82.4 ± 0.6	74.4 ± 1.3
*ℓ*_4_-MKL	**85.2 ± 0.6**	78.5 ± 0.8	82.3 ± 0.6	74.2 ± 1.0
*ℓ*_5_-MKL	85.1 ± 0.6	78.5 ± 0.7	82.3 ± 0.6	74.1 ± 1.3

**Table 3.** Sign test for the performance of MKL algorithms on the METLIN and MassBank datasets

	Acc	UNIMKL	ALIGN	ALIGNF	QCMKL	*ℓ*_2_-MKL	*ℓ*_3_-MKL	*ℓ*_4_-MKL	*ℓ*_5_-MKL
METLIN	UNIMKL		−−	−	++	++		−−	−−
	ALIGN	++			++	++	+		++
	ALIGNF	+			++	++			+
	QCMKL	−−	−−	−−		++	−−	−−	−−
	*ℓ*_2_-MKL	−−	−−	−−	−−		−−	−−	−−
	*ℓ*_3_-MKL		−		++	++			
	*ℓ*_4_-MKL	++			++	++			
	*ℓ*_5_-MKL	++	−−	−	++	++			
MassBank	UNIMKL		−	−−	+	+			+
	ALIGN	+		−−	+	++		++	++
	ALIGNF	++	++		++	++	++	++	++
	QCMKL	−	−	−−			−	−−	−
	*ℓ*_2_-MKL	−	−−	−−				−	−
	*ℓ*_3_-MKL			−−	+			+	
	*ℓ*_4_-MKL		−−	−−	++	+	−		
	*ℓ*_5_-MKL	−	−−	−−	+	+			
	F1	UNIMKL	ALIGN	ALIGNF	QCMKL	*ℓ*_2_-MKL	*ℓ*_3_-MKL	*ℓ*_4_-MKL	*ℓ*_5_-MKL
METLIN	UNIMKL		−			+	−−	−−	−−
	ALIGN	+			++	++			
	ALIGNF				+	++			
	QCMKL		−−	−		+	−−	−−	
	*ℓ*_2_-MKL	–	−−	−−	−		−−	−−	−−
	*ℓ*_3_-MKL	++			++	++			
	*ℓ*_4_-MKL	++			++	++			
	*ℓ*_5_-MKL	++				++			
MassBank	UNIMKL		−						+
	ALIGN	+			++	++			
	ALIGNF				++	++			
	QCMKL		−−	−−			−−	−−	–
	*ℓ*_2_-MKL		−−	−−					
	*ℓ*_3_-MKL				++				
	*ℓ*_4_-MKL				++				
	*ℓ*_5_-MKL	–			+				

‘+’ indicates the method in the row is better than the method in the column (‘−’ otherwise) with significance *P*-value between 0.01 and 0.05; blank indicates no significance. Similarly, ‘++’ and ‘−−’ indicate significance with *P*-value < 0.01. Upper table is for accuracy and lower table is for F1.

### 3.2 Metabolite identification performance

The molecular fingerprint prediction can serve as an intermediate step for metabolites identification, and can be used to search a molecular structure database (Heinonen *et al.*, 2012; Shen *et al.*, 2013). We want to evaluate whether improvements in fingerprint prediction propagate to better metabolites identifications. We will search for molecular structures from the KEGG database. As we assume to know the correct molecular formula, we may filter based on this information to generate our candidate lists. But it turns out that this filter is too strict for a meaningful evaluation, as the number of candidates for each MS/MS spectrum becomes very small and, hence, *all* kernels show good performance. For a more discriminative evaluation of the kernels, we artificially enlarge the set of candidates: we use all molecular structures in KEGG with mass accuracy window $\left[\mu_{M}-\Delta ,\mu_{M}+\Delta \right]$ as candidates, where μ*_M_* is the true mass of the unknown molecule. For sufficiently large mass accuracy Δ, this results in candidate lists that allow a meaningful comparison of the kernels.

**Fig. 2.**
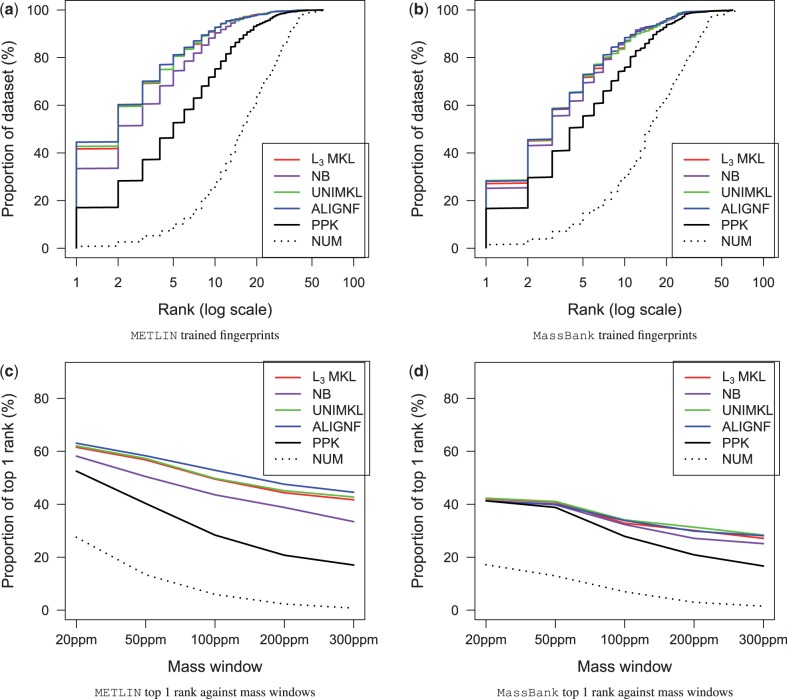
(**a** and **b**) show the performance for identification when searching KEGG using 300-ppm mass window with predicted molecular fingerprints, with fingerprints trained with METLIN and MassBank datasets, respectively. NUM denotes the number of candidate molecules returned per query. (**c** and **d**) show the proportion of data that were correctly identified in the top 1 rank against a series of mass windows

For identification, we want the true molecular structure to be ranked as high as possible in the candidates list. Figure 2a and b shows the fraction of compounds that were ranked higher than certain rank for the two datasets, when searching KEGG with 300 ppm mass inaccuracy to generate the candidates for the two datasets.

We notice that the NB kernel is consistently more accurate than PPK. In addition, MKL clearly improves the identification performance, especially the number of top-ranked identifications increases significantly. *T*-test between the ranks of the ALIGNF and PPK shows a *P*-value of 0.06 which verifies the improvements in identification by ALIGNF over the PPK is indeed significant. ALIGNF comes on top of the MKL approaches, which is in line with its good fingerprint prediction accuracy and F1 score.

The effect of mass accuracy windows during the database retrieval are shown in Figure 2c and d. A narrower 20-ppm mass search window filters out many false candidates, and thus significantly elevates the identification accuracies to 60% on METLIN dataset and 40% on MassBank dataset. However, the effect of improved molecular fingerprint prediction is softened due to the fewer but possibly more similar candidates. An extreme case is observed in Figure 2d in which all the methods shrink to the same result when searching with 20-ppm mass accuracy window.

## 4 DISCUSSION

The present work combines the combinatorial fragmentation tree approach with machine learning through a kernel-based approach. We suggest several kernels for fragmentation trees, and show how to fuse their information through MKL. The result significantly enhances molecular fingerprint prediction and metabolite identification.

The closest analogs to our fragmentation tree kernels in literature are those defined for parse trees in natural language processing (Collins and Duffy, 2001); our fragmentation trees can be seen as parses of the MS/MS spectra. DP techniques similar to ours are used there for computing kernels between trees (Collins and Duffy, 2001; Kuboyama, 2007). However, fragmentation trees have important differences to the trees defined between parses of natural language and to kernels comparing molecular structures (Mahé and Vert, 2009). Differently from natural language parses, the node labels have partial order (via their molecular weights) and also the edges have labels. Differently from kernels for molecular graphs, the label spaces of both nodes and edges are vast (subsets of molecular formulae).

The comparison with the PPK employed by the FingerID (Heinonen *et al.*, 2012) software shows that the fragmentation tree kernels are able to extract more information out of the MS/MS spectra. Improvements are seen in both the prediction accuracy and the F1 score. Comparing with FingerID (PPK), the uniform combination of the kernels (UNIMKL) improves the molecular fingerprint prediction significantly in accuracy and F1. As witnessed by many MKL applications, the UNIMKL algorithm is hard to beat. In our result, several MKL algorithms such as ALIGNF and *ℓ*_3_-norm can give slightly better result than UNIMKL. The improvements in the molecular fingerprint prediction translate to improved metabolite identification.

There are several possible routes forward with the current metabolite identification framework. First, post-processing on the candidates list, such as the one proposed by Allen *et al.* (2013), is necessary when searching a large compound database such as PubChem, because the returned candidates (hundreds to thousands) may share the same fingerprints and there is no way to differ them based only on molecular fingerprints. Second, training a separate SVM for each fingerprint property is clearly an aspect that can be improved upon, for example, by a multi-label classification approach. A still more tempting yet challenging direction would be to replace the two-step identification by an integrated prediction approach. Such an approach would potentially learn to predict the fingerprint properties that are important for discriminating metabolites from each other.

*Funding*: Academy of Finland grant 268874 (MIDAS); Deutsche Forschungsgemeinschaft grant (BO 1910/16-1) (IDUN). 

*Conflict of Interest*: none declared.
